# Editorial: Consequences of the COVID-19 Pandemic on Care for Neurological Conditions

**DOI:** 10.3389/fneur.2021.788912

**Published:** 2021-11-15

**Authors:** Jordi A. Matias-Guiu, Sheng-Feng Sung, Cheng-Yang Hsieh, Tomohisa Nezu, Jesús Porta-Etessam, Ricardo F. Allegri

**Affiliations:** ^1^Department of Neurology, Hospital Clínico San Carlos, Health Research Institute “San Carlos” (IdISCC), Universidad Complutense de Madrid, Madrid, Spain; ^2^Division of Neurology, Department of Internal Medicine, Ditmanson Medical Foundation Chia-Yi Christian Hospital, Chiayi City, Taiwan; ^3^Department of Neurology, Tainan Sin Lau Hospital, Tainan, Taiwan; ^4^Department of Clinical Neuroscience and Therapeutics, Hiroshima University Graduate School of Biomedical and Health Sciences, Hiroshima, Japan; ^5^Department of Cognitive Neurology, Neuropsychiatry and Neuropsychology, Instituto Neurológico Fleni, Buenos Aires, Argentina

**Keywords:** COVID-19, SARS-CoV-2, neurological care, coronavirus, healthcare

The coronavirus disease 2019 (COVID-19) pandemic has caused a wide range of unprecedented consequences, including social, economic, and health disruptions. From the point of view of healthcare assistance, COVID-19 has deeply impacted usual practice at all levels since the beginning of 2020. In this setting, neurological assistance has adapted to the circumstances of the pandemic. In fact, because COVID-19 involves neurological symptoms, affected patients require the attention of neurologists, and the high demand for clinical care entailed the recruitment of many neurologists to frontline assistance (1). In addition, the pandemic has impacted the management of patients with neurological disorders, with changes in the management of relapses, usual follow-up, diagnostic procedures, implementation or generalization of telemedicine, etc. Lockdown and social isolation were also very harmful in patients with neurological disorders (2). Furthermore, the treatment of neurological emergencies, such as stroke, was also compromised because of resource re-allocation during the emergency, and the fear of patients to attend the hospital.

The neurological community needed to share experiences about how to face this global challenge. Accordingly, this Research Topic was launched in April 2020 to address these issues. Over 117 manuscripts were submitted, and 76 papers have been published, including originals, reviews, and case reports. Studies have covered the main areas of neurological care, including general neurological care, stroke, epilepsy, multiple sclerosis, movement disorders, cognitive neurology, neuromuscular disorders, headache, and neuropediatrics.

## Studies on General Neurological Care

Healthcare systems were challenged by the COVID-19 pandemic. In a study by Calandri et al., emergency department and outpatient consultations to a tertiary neurological center during the first wave in Argentina were significantly correlated with social mobility estimated by the Google Mobility Index as a result of the long lockdown. Besides, telemedicine was successfully implemented, with increased access to distant zones, which may be important for better access to specialized neurological care.

Protocols of changes in neurological care were proposed in two studies conducted by the Pandemic Health System Resilience Program Consortium (REPROGRAM) Pathway in both the acute and chronic settings (Bhaskar, Sharma et al.; Bhaskar, Bradley et al.). These changes were considered appropriate to ensure healthcare professionals' and patients' safety and minimize the impact of the pandemic in neurological care.

## Studies on Stroke and Neurocritical Care

Stroke was one of the main subjects addressed in this Research Topic, with a large number of articles. Many regions reported a decline in stroke admissions during the early months of the pandemic. This reduction was mainly observed in the most severe and disabling strokes (Yao et al.), and was reported in several countries and settings, and for ischemic and hemorrhagic stroke, especially during the lockdowns (Abdulazim et al.; Ota et al.; Uphaus et al.; Erdur et al.). Conversely, the impact on the reperfusion procedures was variable, although it was also affected in the peak of the pandemic as suggested by several studies (D'Anna et al.; Koge et al.).

Some papers proposed modifications in the protocols for stroke care, from the acute phase to the rehabilitation therapies, suggesting a complete reorganization of the assistance to avoid the worsening of outcomes of stroke patients [Venketasubramanian; Candeloro et al.; Al-Jehani et al.; (3)].

Other studies focused on the relationship between COVID-19 and stroke, contributing to the knowledge about stroke subtypes, severity, management, and outcomes (Fraiman et al.; Grewal et al.; Tiwari et al.; Yang et al.; Wang et al.).

## Studies on Epilepsy

The treatment of patients with epilepsy is another of the topics examined in the Research Topic. Mostacci et al. reviewed the literature regarding the impact of the pandemic on the health of patients with epilepsy, and conducted a survey regarding this topic. Although most patients did not report a significant change, clinical worsening was detected in a proportion of changes and was associated with sleep disorders or limited access to healthcare, among other factors. Lanzone et al. conducted a study evaluating the impact of lockdown in patients with epilepsy. In this case, the survey was analyzed with a text mining approach. Patients with epilepsy used different kinds of words, suggesting a different reaction to the lockdown compared with controls, which may be important in the follow-up and treatment of these patients during traumatic or stressful events.

Telemedicine has become very widespread in epilepsy care since the pandemic. Accordingly, a decision-making tree to manage patients with epilepsy was proposed (Kuroda).

## Studies On Multiple Sclerosis

Patients with multiple sclerosis (MS) were regarded as a high-risk population due to the potential immunosuppressive effect of several treatments used in the disease. In a study by the Portuguese Multiple Sclerosis Study Group a consensus was obtained using a Delphi methodology about the implementation of several changes in the management in the context of the pandemic (Cerqueira et al.).

## Studies On Parkinson's Disease

Several studies evaluated the impact in patients with Parkinson's disease and other movement disorders of the pandemic. Hanff et al., used a semi-structured interview to examine unmet needs during the lockdown, and Piano et al., examined the impact of lockdown in patients treated with deep brain stimulation. Motolese et al., reported the experience of a remote monitoring program during the lockdown, with adequate degrees of satisfaction. Similarly, Shalash et al., presented their experience of virtual visits in patients with Parkinson's disease in Egypt.

## Studies On Cognitive Neurology

Patients with dementia were among the most affected by both the quarantine and COVID-19 (LaHue et al.). The effects of lockdown resulted in a clinical worsening in patients with mild cognitive impairment and dementia (Barguilla et al.; Pelicioni et al.). In addition, cognitive training and rehabilitation were adapted to telehealth. In this regard, Bernini et al. implemented HomeCoRe, an innovative approach to offer remote cognitive training.

## Studies On Neuromuscular Disorders

Patients with neuromuscular disorders were at higher risk of COVID-19 due to the frequent respiratory involvement and some immunosuppressive therapies. In this regard, several studies evaluated the impact of the pandemic and the SARS-CoV-2 infection in patients with neuromuscular disorders. Katyal et al., reviewed the potential neuromuscular complications of COVID-19, such as Guillain-Barre syndrome. In two studies by Bertran Recasens and Rubio and Tseng and Chen, the authors proposed recommendations for the management of patients with neuromuscular disorders during the pandemic or during COVID-19, and evaluated the impact of the pandemic on these patients.

## Studies On Headache

Headache is one of the most frequent symptoms during COVID-19. However, primary headaches are also influenced by environmental factors. This topic was examined by Delussi et al., in a cross-sectional study from the Italian National Headache Registry, in which changes in migraine during the quarantine were investigated. According to the study by Dallavale et al., in children and adolescents, there was a mild improvement of migraine symptoms. Furthermore, Planchuelo-Gómez et al., tried to characterize the headache phenotypes in COVID-19 and link these features with inflammatory biomarkers.

## Studies On Neuropediatrics

SARS-CoV-2 also affects children, although to a lesser extent than adults. In the article by Boronat, the evidence about neurological symptoms and complications in children was reviewed. Compared with adults, neurological complications were less frequent. However, a multisystem inflammatory syndrome can occur, with the development of encephalopathy as the most frequent clinical manifestation. Furthermore, in this article, a review of the changes in the care of children with neurological disorders (e.g., cerebral palsy, epilepsy, attention deficit and hyperactivity disorder, etc.) was presented. Other collateral problems in children have been the closure of schools, lack of free time outdoors, and financial insecurity.

## Studies on Diagnostic Procedures

Another important challenge during the pandemic concerns the use of diagnostic tools. Postponed examinations are associated with diagnostic delays and worse outcomes. Currently, these techniques are key for adequate neurological care, but they may entail a risk of COVID-19 infection. In this regard, González-Ortiz et al., Chen et al., and Valsamis et al. proposed process modifications for neuroimaging, cerebrospinal fluid samples, and electroencephalography.

## Conclusions

In this Research Topic, the authors have presented important experiences and solutions to the changes generated by the COVID-19 pandemic in neurological care. Most of the studies were conducted during the first stages of the COVID-19 to face many of the challenges that had arisen, including the lockdown and quarantines, safety concerns because of high transmissibility, and risks of COVID-19 in patients with neurological disorders. As in other crises, the lessons learned should be applied for the future benefit of patients and the healthcare system during the COVID-19 pandemic, and probably afterward. Some changes in neurological care, such as safety protocols or implementation of teletherapy, may be helpful and relevant in daily practice (4, 5). How the current pandemic will give rise to long-term changes in neurological assistance, and the convenience of these changes, should be evaluated in the future.

## Author Contributions

JM-G wrote the first draft of the manuscript. RA wrote parts of the manuscript. All authors contributed to manuscript revision, read, and approved the submitted version.

## Conflict of Interest

The authors declare that the research was conducted in the absence of any commercial or financial relationships that could be construed as a potential conflict of interest.

## Publisher's Note

All claims expressed in this article are solely those of the authors and do not necessarily represent those of their affiliated organizations, or those of the publisher, the editors and the reviewers. Any product that may be evaluated in this article, or claim that may be made by its manufacturer, is not guaranteed or endorsed by the publisher.
